# What should medical students do to choose their specialty?

**DOI:** 10.11604/pamj.2015.22.282.7955

**Published:** 2015-11-23

**Authors:** Omar Ali Aboshady, Maha Saad Zenhom, Abdelrahman Ashraf Nasr

**Affiliations:** 1 Faculty of Medicine, Menoufia University, Menoufia, Egypt; 2 Faculty of Medicine, Ain shams University, Cairo, Egypt

**Keywords:** Career choice, medical students, specialty

## Editorial

Career choice is both an important and inevitable milestone in the life of each medical student. Such a choice can have a great impact on a given students future plans such as life goals as well as their family and social life. Choosing a well-fitting specialty can boost students’ career achievements and enhance their quality of life. Moreover, it can directly affect the healthcare system by causing either a shortage or oversupply of available physicians in different specialties, which can lead to an unbalanced availability of health-care services [1]. While the career-choice stage of a student's life was expected to be filled with enthusiasm and optimism, it was instead found to be one of the most critical and stressful times due to the complex, dynamic and multifactorial nature of the decision-making process [2]. The 2015 report of The Association of American Medical Colleges (AAMC) on Residents reported that 56 percent of medical students change their preferred residency specialty prior to the completion of medical school [3]. This may be attributed to the poor orientation and lack of career-support services provided to many students. This usually leads to inappropriate career selection and even a career change a few years into training. Studies have shown that 20 percent of residents [4] and 16 percent of physicians change their specialties to completely unrelated fields [5]. Concerning practice, medical specialties can be classified into two categories: 1) person-oriented specialties, such as family medicine, internal medicine and pediatrics; and 2) technique-oriented specialties, such as surgery, pathology and anesthesiology [6]. Selecting a career choice among these specialties usually depends on different intrinsic (i.e. related to personal attributes and preferences) and extrinsic (i.e. related to work environment) factors [2]. These include personal fit, gender, controllable lifestyle, previous clinical experience, role model effect, financial reward, prestige, work pressure, future job security, working hours, early career training quality, subspecialty choice and nature of patient care [2, 7, 8]. In this editorial, we highlight five important tips that may help students to better choose their careers.

## Explore yourself

Personal fit is considered the most influential factor in choosing a medical specialty. It results from exploring three important variables: one's self, one's peers within the same specialty and the nature of practice in the chosen specialty [9]. In other words, it means to exclude specialties that do not present you with enough excitement, challenge, control and pleasure. While considering which specialty to choose, keep in mind that finding the right specialty for yourself will depend on an examination of these three variables. Some personality tests would help you to better identify your preferences such as Myers-Briggs Type Indicator [10] or Zuckerman-Kuhlman personality questionnaire-medium form [11]. For each specialty you consider, you may end up confirming pre-existing beliefs about that specialty, eliminating it as an option, or grouping it with other choices and seeking a more general specialty [9]. However, it is important to make your choice represent a compromise between your interests, capacities, values as well as the available opportunities and their limitations [6].

## Research and compare specialties

To better compare between specialties, one must ask themselves a few questions: What is the type of this specialty (general or special)? What types of patients do you prefer? How much contact with patients do you want? What is the intellectual content of this specialty? What kind of lifestyle do you prefer? What is the prestige and social expectation do you want? What is the length of residency? What are the educational opportunities after finishing residency? What are the job opportunities of this specialty? How will be the future income and economic stability? The way to reach these answers is hard and requires to begin researching from the first year of medical school. You can find some help through Internet and some supportive programs like "Careers In Medicine" [12] or "Glaxo Pathway Evaluation Program" [13] that help you to undergo the specialty selection process. However, attending clinical rotations, grand rounds and national and international conferences, that help interact directly with relevant resources, is the best way to identify career information. Specialty interest groups may be also of help. If you remained undecided after collecting all these information, performing a research for a period of time is the best way to help you convince your choices [14].

## Consider factors that may affect your decision

From literature, there are certain factors that may affect your career choice. For example, gender may be considered the natural selector of specialties due to the wide variance between males and females in their choices. It has been reported in multiple studies that males usually prefer surgical specialties, in contrast to the females who prefer the medical ones [7]. In addition, cultural and religious backgrounds may play a role in preferring some specialties such as obstetrics and gynecology, especially in Islamic countries [15]. Social and economic factors are also important factors that may affect your decision [16]. Therefore, it is important to study the effects of these factors and plan how you will manage them.

## Engage in clinical experiences

Multiple studies have showed that clinical experience can affect career choice of medical students [14]. Attending elective clerkships or other clinical activities can help you experience what day-to-day clinical life is like in different specialties and help you discover your abilities and skills better [9]. This will help cultivate your interests and start narrowing down precisely your specialty choices. In addition, it will help you view specialties from a more practical perspective, make a more informed choice, and help you to make an early career decision, which could give you much more time to develop your skills and experience in that particular career.

## Do career counseling

Seeking counseling from role models or university counseling services may help you test whether what you have perceived through your investigation matches reality or not. Attending educational activities for career planning and how to choose your career will help you get a clearer picture of the decision-making process [15], decrease stress and confusion, and make you more aware of career options that you might have not considered before. However, it is important not to over counsel. You should seek advice with an aim in mind and not just as a relief.

## Conclusion

The road to a well-fitting specialty is not easy as it may seem; it is full of difficulties and obstacles for every medical student. Changing your career preferences with time is common and natural during medical school [16]. However, you should make a real effort to reach your best choice early in medical school. You should discover your identity by challenging yourself in different situations and specialties. In addition, you should not make a decision based solely on advice, thinking or guessing; instead, try to make a well-informed educated decision by employing all the available tools.
